# Supplementary material for: Longitudinal ridges imparted by high-speed granular flow mechanisms in martian landslides
Source: Nat Commun. 2019 Oct 24;10:4711. doi: 10.1038/s41467-019-12734-0 (PMC6813353; doi:10.1038/s41467-019-12734-0)
Supplement: Supplementary file 1 — Supplementary Information [file 41467_2019_12734_MOESM1_ESM.pdf]

SUPPLEMENTARY INFORMATION

"Longitudinal ridges imparted by high-speed granular flow mechanisms in martian landslides"

by Magnarini et al.

Supplementary Figures.

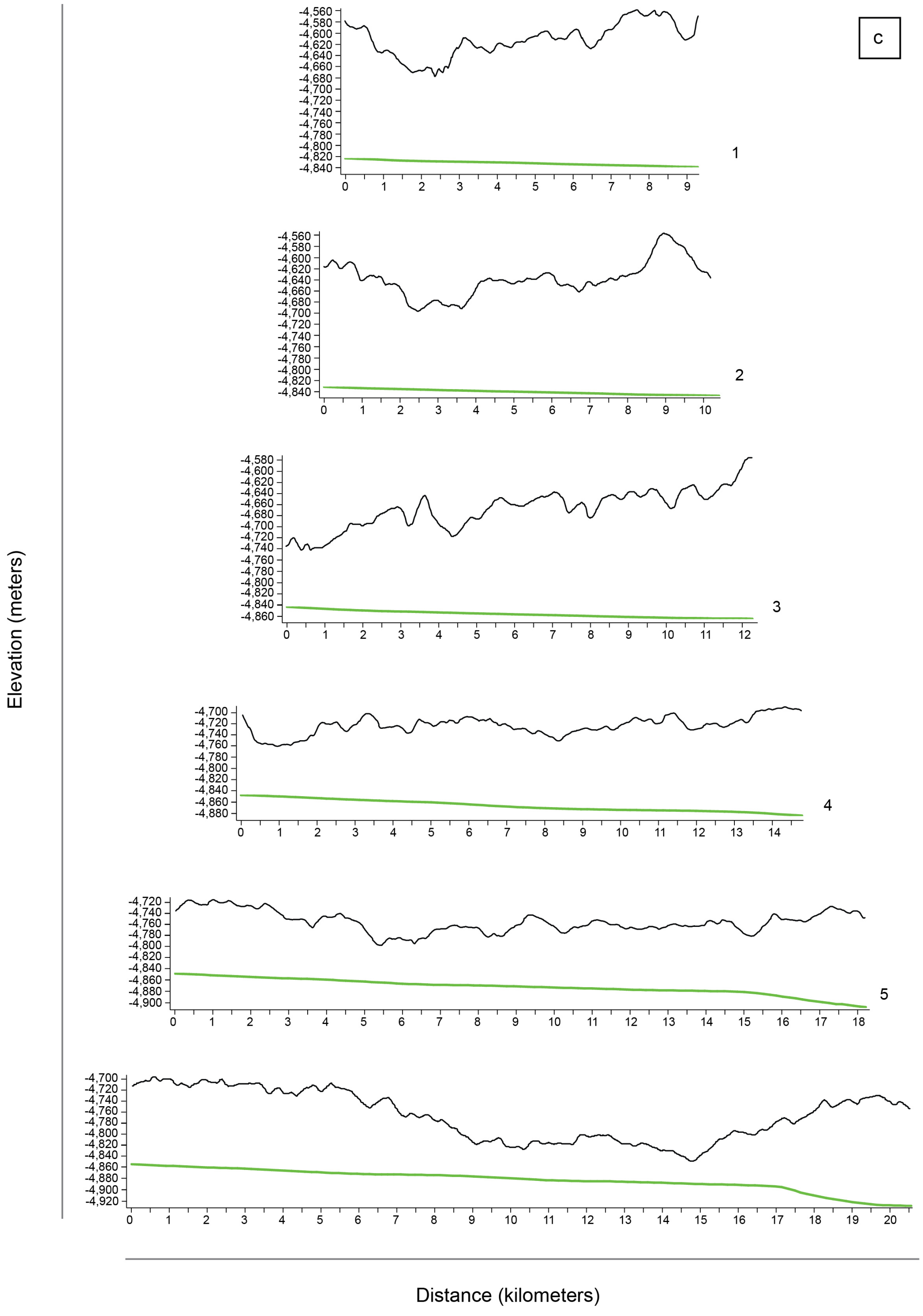

Supplementary Figure 1| Study area (c) (as in Figure 1, main text). Topographic profiles of the landslide deposit (black lines) and topographic profiles of the valley floor (green lines).

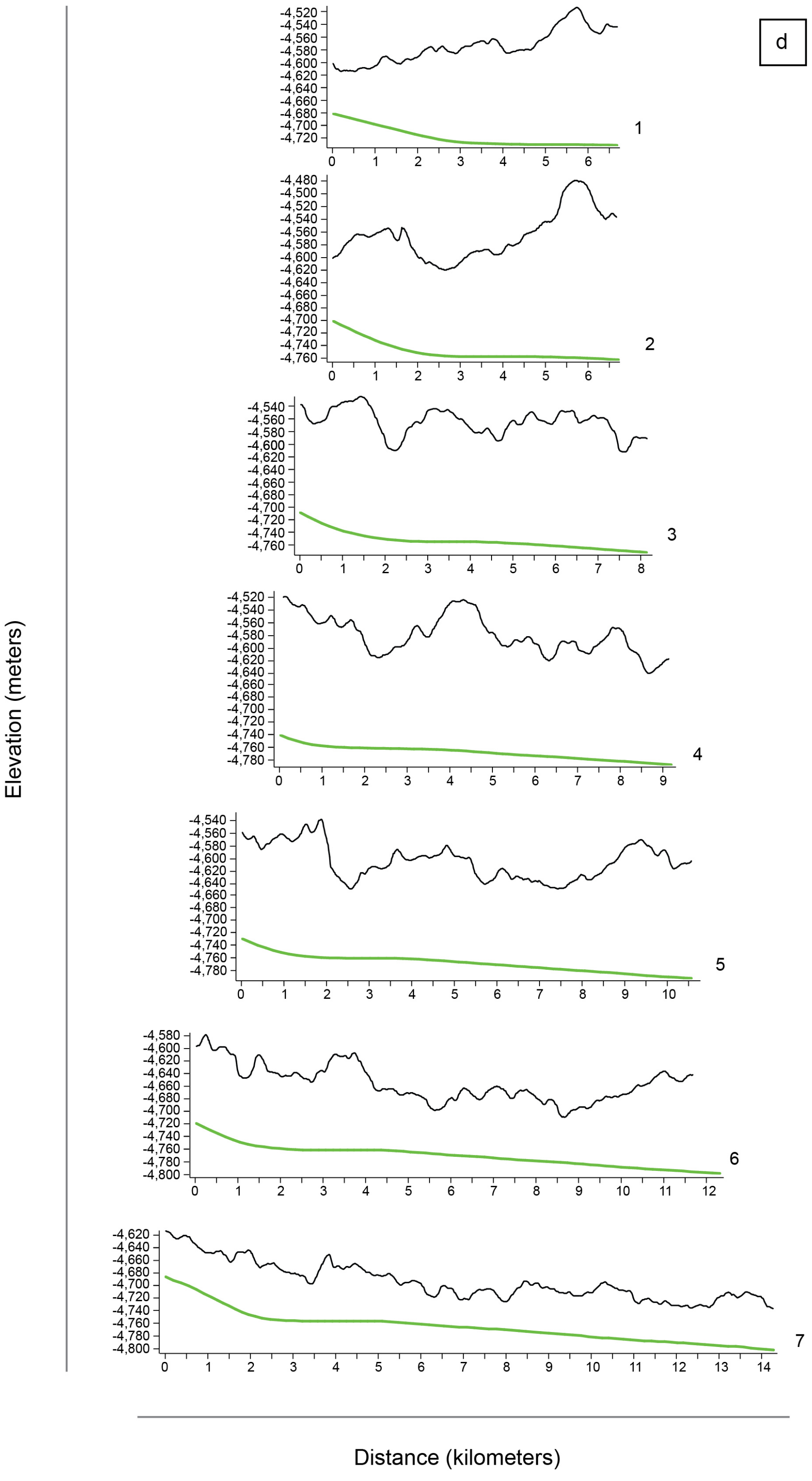

Supplementary Figure 2| Study area (d) (as in Figure 1, main text). Topographic profiles of the landslide deposit (black lines) and topographic profiles of the valley floor (green lines).

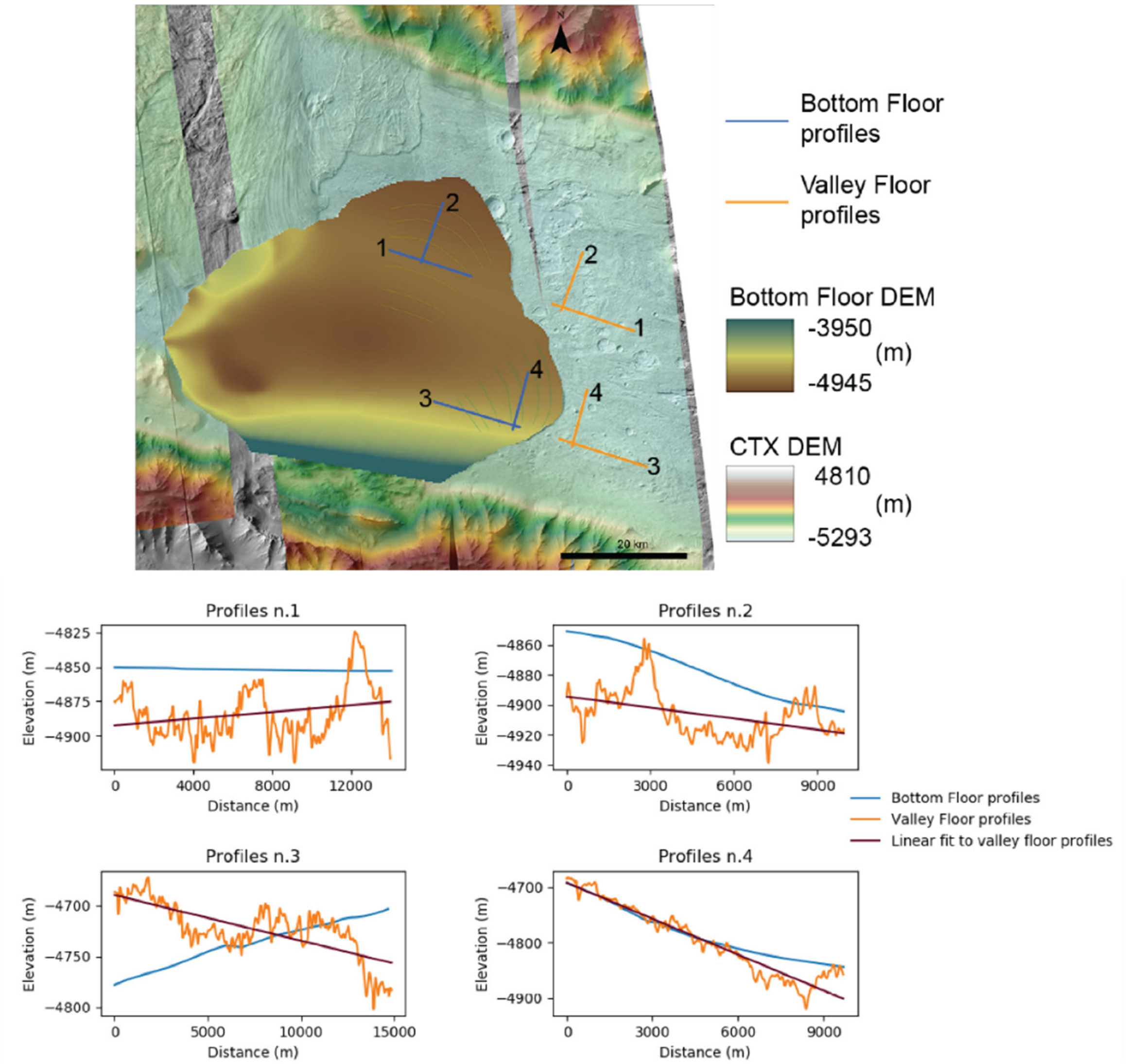

Supplementary Figure 3 | Top figure shows the reconstructed DEM of the valley floor underneath the landslide deposit as inferred by interpolation of the CTX DEM contour lines adjacent the deposit. Plots are showing the comparison of mirror transects (blue and yellow lines 1-4).

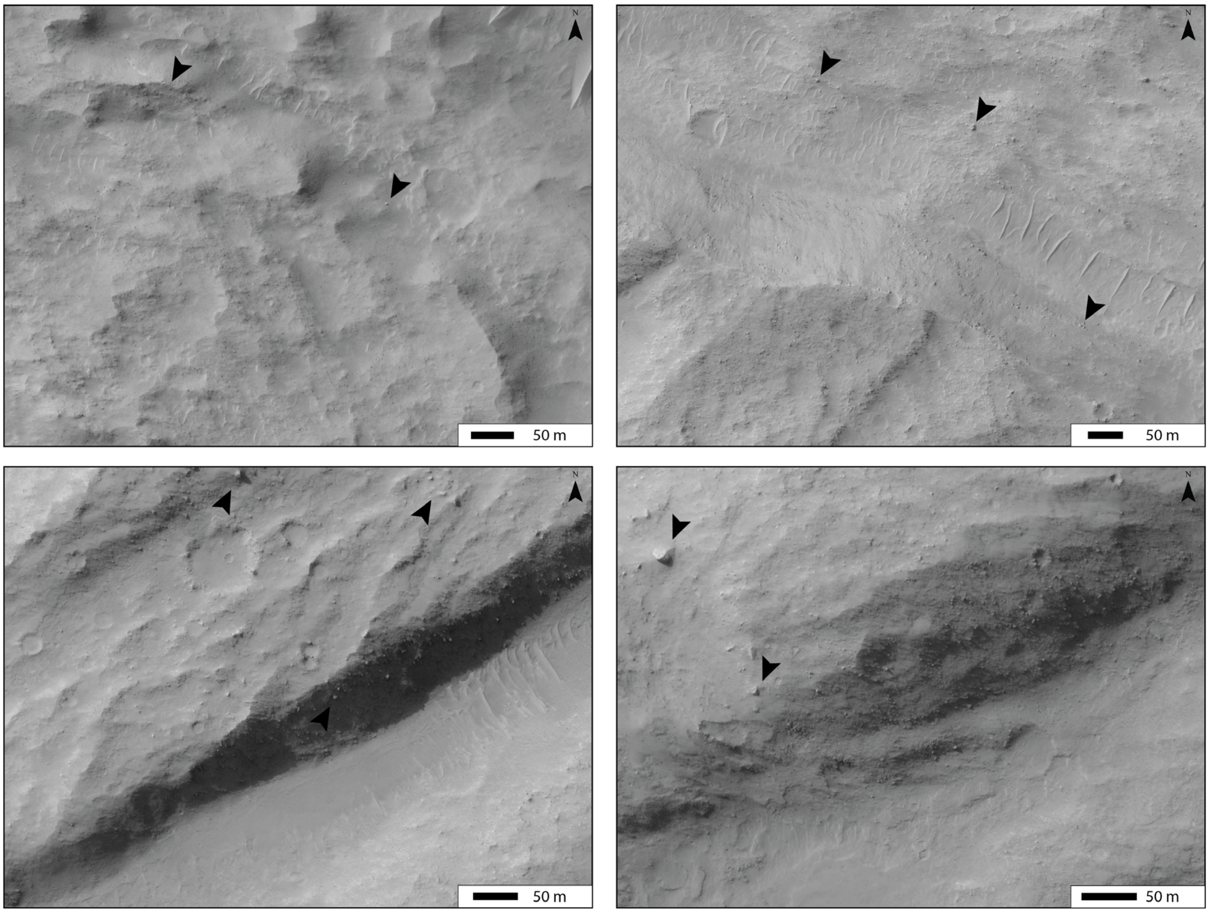

Supplementary Figure 4| Close-up images showing the range of grain sizes, spanning from 1 m (minimum size recognizable given image resolution) up to 20 m (HiRISE image PSP_008906_1685). Note that the grain size we use may be not representative of the actual size range, as biased by resolution limitation and by what is observable at the surface, as it is well-known that in large landslide deposits the largest clasts usually work their way to the top of the deposit, with finer material below. In such a case, the size range of surface boulders may exaggerate the representative grain size. The Frank Slide is a well-known terrestrial case of long runout landslide and its debris was well-described by Cruden and Hungr (1986). The authors indeed observed vertical sorting within the deposit and accumulation of large, predominantly angular boulders in the upper part of the debris. Based on these observations they concluded that the inverse grading indicates the slide was not fluidized by gas pore

pressures and that the boulders were probably supported by dispersive forces and motion-induced vibration. The model that we adopt from experimental granular flow studies[19,20] also suggests the idea that there are vertical movements of material during the landslide propagation by means of convective longitudinal cells. Our observations of boulders at the surface of the Martian landslide add to this idea.

<u>**Supplementary Tables.**</u>

Central Study Area (c)

Profile	N. of ridges	Density of ridges (m^{-1})	Profile Length (m)	(S) Average Spacing (m)	(T) Average Thickness (m)	S/T
1	15	1.6	9283	607	222	2.7
2	18	1.7	10409	562	205	2.74
3	26	2.1	12225	465	182	2.55
4	40	2.6	15134	368	143	2.56
5	51	2.8	18105	353	118	2.99
6	63	3	20809	329	114	2.87

Supplementary Table 1 | Results for Study Area (c).

Eastl Study Area (d)

Profile	N. of ridges	Density of ridges (m^{-1})	Profile Length (m)	(S) Average Spacing (m)	(T) Average Thickness (m)	S/T
1	16	2.5	6449	413	170	2.4
2	17	2.5	6862	400	185	2.17
3	23	2.8	8110	352	192	1.83
4	33	3.6	9146	277	193	1.44
5	39	3.7	10575	272	169	1.6
6	49	3.9	12409	253	110	2.3
7	82	5.7	14339	176	73	2.4

Supplementary Table 2 | Results for Study Area (d).

Image Pairs (ID number)	Pixel size (m)	Incidence angle (o)	Phase angle (o)	Emission angle (o)	IFOV (m)	Vertical Precision (m)
B21_017688_1685_XN_11S067W	5.24	60.49	63.02	3.61	5.66	2.35
B22_018321_1685_XN_11S068W	6.06	62.54	47.41	22.17		
P20_008906_1685_XN_11S067W	5.28	61.93	57.0	19.47	5.56	2.24
P22_009763_1690_XN_11S067W	5.82	60.58	76.01	26.4		
P19_008616_1689_XI_11S068W	5.28	60.41	58.16	15.83	5.45	3.18
P21_009051_1688_XN_11S068W	5.61	60.79	72.57	18.89		
P16_007113_1678_XN_12S067W	5.25	45.61	49.76	8.6	5.29	13.08
F20_043784_1677_XI_12S067W	5.32	58.72	452.86	4.62		

Supplementary Table 3 | List of CTX image pairs used to make DEMs. Vertical precision is estimated using the method of Okubo (2010).

Profiles	Standard deviation σ	Propagation of error σ_k
Yellow line 1	18.98 m (σ_i)	24.61 m (Central study area)
Yellow line 2	15.67 m (σ_j)	
Yellow line 3	25.86 m (σ_i)	67.9 m (East study area)
Yellow line4	62.78 m (σ_j)	

Supplementary Table 4 | Standard deviation of the topographic elevation measurements along each set of transects and propagation of error, as derived from Equation 1, to evaluate errors on the deposit thickness calculation for the central area of study (c) and the east area of study (d).

Supplementary References.

Cruden, D. M. & Hungr O., The debris of the Frank Slide and theories of rockslide-avalanche mobility. Can. J. Earth Sci. **23**, 425-432 (1986).

Okubo, C. H., Structural geology of Amazonian-aged layered sedimentary deposits in southwest Candor Chasma, Mars. Icarus **207**, doi:10.1016/j.icarus.2009.11.012 (2010).
